# Efficacy and acceptability of a blended intervention for emotion regulation (MAISHA) among young people in Kenya: study protocol for a cluster RCT

**DOI:** 10.1186/s40359-025-03837-z

**Published:** 2025-12-20

**Authors:** Esther Rugendo, Glorialoveness S. Lyimo, Rahim Daya, Moreen Githinji, Kevin Kashamba, Faith Njoki, Innocent Yusufu, Tom Osborn, Rachel P. Chase, Mary Sando, Azan Nyundo, Till Bärnighausen, Ulrich Reininghaus, Shannon A. McMahon, Annika Stefanie Reinhold

**Affiliations:** 1 Heidelberg Institute of Global Health, Heidelberg University, Im Neuenheimer Feld 130.3, Heidelberg, 69120 Germany; 2 Shamiri Institute, Nairobi, Kenya; 3 Department of Clinical Nursing, University of Dodoma, Dodoma, Tanzania; 4 Africa Academy for Public Health (AAPH), Dar es Salaam, Tanzania; 5 Department of Public Mental Health, Medical Faculty Mannheim, Central Institute of Mental Health, Heidelberg University, Mannheim, Germany; 6 Health Service and Population Research Department, Institute of Psychiatry, Psychology & Neuroscience, King’s College London, London, UK; 7 German Center for Mental Health (DZPG), partner site Mannheim- Heidelberg-Ulm, Heidelberg, Germany

**Keywords:** Cluster RCT, Emotion regulation, Young people, Kenya, Blended care, Digital app, Micro-randomized trial, Mental health, Low-and middle-income countries

## Abstract

**Background:**

Adolescence is a crucial developmental period characterized by increased challenges in emotion regulation, a key mechanism linked to mental health. In Kenya, one in every two adolescents and youth faces a mental health challenge, highlighting a need for early interventions targeting emotion regulation. This study will evaluate the efficacy and acceptability of MAISHA, a blended indicated mental health intervention co-designed with young people to strengthen emotion regulation among those experiencing related difficulties.

**Methods:**

In a parallel group, cluster-randomized 2 × 2 factorial controlled trial, 666 young people aged 13–25 will be randomly allocated to one of four 4-week study arms at a ratio of 1:1:1:1. (1) digital app blended with a face-to-face element; (2) digital app only; (3) face-to-face element only; or (4) care as usual (CAU). The digital app will build on an ecological momentary intervention. The face-to-face element will consist of four group sessions on growth mindset, values, and gratitude. The primary outcome is emotion regulation difficulties, assessed post-intervention using the short form of the Difficulties in Emotion Regulation Scale (DERS-SF), and comparing the blended intervention (digital and face-to-face) to CAU. Secondary measures include emotional reactivity, emotional distress, positive and negative affect, acceptability, desirability, safety, and usability. The trial will include an embedded micro-randomized trial and a process evaluation.

**Discussion:**

This trial advances adolescent global mental health by strengthening evidence on youth-focused, mechanism-informed, blended interventions while also highlighting participatory approaches in LMICs.

**Trial registration:**

The trial was registered in the Pan African Clinical Trials Registry on July 17th, 2025, as PACTR202507903096871 (https://pactr.samrc.ac.za/TrialDisplay.aspx?TrialID=34896).

**Supplementary Information:**

The online version contains supplementary material available at 10.1186/s40359-025-03837-z.

## Background

Worldwide, mental health conditions account for the largest burden of disease among adolescents and youth aged 10–25 (13%), threatening their well-being, survival, and future prospects [1]. In low and middle-income countries (LMICs), this age group faces disproportionate levels of mental health conditions due to widespread poverty, violence, stigma, and limited and unaffordable mental health services [2–4]. In sub-Saharan Africa (SSA), youth and adolescents constitute the greatest proportion of the population (33%) [5], yet one in seven individuals suffers from major psychological distress [6]. A recent meta-analysis revealed that 14.5 years of age is the peak risk period for the onset of mental conditions, some of which persist into adulthood [7]. In Kenya, the prevalence of depression and anxiety among youth and adolescents is 44% and 38%, respectively [3, 8], yet access to evidence-based care remains limited. Care as usual typically involves informal support from family members or peers. Standard public sector services for mental, neurological, and substance use disorders exist. However, these services are often constrained by a shortage of health care workers and low mental health literacy, resulting in gaps in accessibility and continuity of care [9, 10]. Locally developed community-based approaches working with lay providers aim to complement CAU, but are not yet part of established care pathways [11].

Adolescence is a crucial developmental period characterized by numerous physical, cognitive, and emotional changes. Some young people respond to these challenges by strengthening their emotion regulation (ER) skills, whereas others experience ER difficulties [12, 13], which are associated with a greater risk of developing mental health conditions [14, 15].

ER – defined as “the process by which individuals influence which emotions they have, when they have them, and how they experience and express them” [13] – is recognized as a transdiagnostic risk and resilience mechanism affecting many common mental health conditions [16–18]. In a recent meta-analysis [19] and a global systematic review [20], ER was identified as a central mechanism of change and the core therapeutic component of effective mental health interventions for young people. Strengthening ER improves mental resilience, emotional well-being, problem-solving, behavior change, and academic performance [21, 22]. These insights underscore a need for ER interventions that can promote mental health among young people.

The choice, utility, and effectiveness of ER strategies depend on situations and contexts [23], with emotions fluctuating within a single day [24]. To capture this dynamic nature of emotions and intervene in real-time, ecological momentary assessment (EMA) [25] and ecological momentary interventions (EMIs) [26] are particularly well-suited approaches. EMA involves collecting data repeatedly throughout the day, capturing participants’ emotional states, behaviors, and context in their natural environments. EMI delivers adaptive interventions in response to EMA data, targeting specific moments [26] when a participant is experiencing emotional distress or could benefit from support. For example, when an adolescent reports high stress or negative affect in an EMA, the app may prompt a short reflection or breathing exercise to help regulate emotions in real time [26]. Preliminary findings from an exploratory randomized controlled trial (RCT) of a mobile EMI addressing ER in young people with early mental health problems [27, 28] suggested a reduction in emotional reactivity. We build on this mobile EMI, originally developed and evaluated in Germany, as a foundation for the digital element of our intervention.

While EMIs and other digital mental health interventions have demonstrated potential to increase access to services, digital-only approaches may lack the interpersonal connection offered by face-to-face formats. Blended care models, which combine digital and face-to-face intervention elements, offer a promising solution to provide more flexible and inclusive mental health support [29]. Blended interventions combine the benefits of both approaches, can enhance the quality of mental health services, and are acceptable among young people [28, 29]. To complement the digital element of our intervention, we integrate it with a Kenyan caregiving model that is effective in reducing depression and anxiety symptoms [30]. Integrating EMI into the face-to-face model allows for timely and personalized support in an adolescent’s daily life beyond the group setting. The digital element thereby extends practice into real-world contexts, and may increase scalability through mobile delivery, particularly in low-resource settings. Both the digital and face-to-face elements form the full blended “MAISHA” intervention.

Research demonstrates that culturally adapted interventions can enhance user engagement, increase acceptability, and usability [31] and ultimately improve effectiveness [32]. For young people in particular, intervention development should also consider their socio-cultural contexts and needs. Building on this, we adapted MAISHA with young people using human-centered design [33], and we will further involve them in the trial through a youth participatory action research (YPAR) approach [34].

This study aims to test the efficacy and acceptability of MAISHA, a culturally adapted blended intervention to improve ER, through a cluster-randomized 2 × 2 factorial RCT conducted among young people aged 13–25 in Kenya. The primary outcome is emotion regulation difficulties, assessed post-intervention using the short form of the Difficulties in Emotion Regulation Scale (DERS-SF) and compared between the blended MAISHA intervention and CAU.

### Goals and hypotheses

This study has three specific goals:

1. Test the efficacy of the blended intervention, MAISHA, on ER difficulties (primary outcome) among young people in Kenya. We hypothesize that participants allocated to the blended intervention condition (digital + face-to-face) will exhibit lower levels of ER difficulties compared to those in the control condition (CAU) at the post-intervention stage.
2. Examine the signals of efficacy of the blended intervention on secondary mental health outcomes. We hypothesize that participants assigned to the blended intervention will show higher positive affect, more adaptive emotion beliefs, and greater use of adaptive ER strategies, as well as lower negative affect, emotional reactivity, and symptoms of depression, anxiety, and stress compared to CAU. We will also compare the three intervention conditions with each other to explore the potential contribution of intervention elements to the overall effects.
3. Assess the acceptability, desirability, usability, and safety of the blended mental health intervention components. We postulate that the blended intervention will be culturally acceptable, desirable, usable, and safe for participants.
4. Investigate proximal signals of efficacy of momentary intervention components of the digital element. We hypothesize that these intervention components will lead to short-term improvements in momentary negative affect and ER difficulties. Using a micro-randomized trial nested within study arms that include the digital element, we will also explore when, under what conditions, and for whom specific components are effective.

## Study methods

### Study design and setting

This protocol was developed following the SPIRIT 2025 Statement [35] (see Additional File 1: Supplementary methods 5) and will be reported in line with CONSORT 2025 guidelines [36]. In a 2 × 2 parallel group, cluster-randomized controlled factorial trial design, young people aged 13–25 with difficulties in ER will be randomly assigned at a ratio of 1:1:1:1 to one of four conditions: (A) the full blended MAISHA intervention, combining the digital with the face-to-face element; (B) the digital app only; (C) the face-to-face element only; or (D) broadly defined care as usual (CAU). Participants will be recruited from high schools, universities, and community-based organizations for out-of-school individuals. Randomization will be conducted off-site using a computer-generated sequence, and outcome assessment and statistical analyses will be blinded. The primary comparison will assess the superiority of the full blended intervention (A) over CAU (D) in reducing ER difficulties. Exploratory comparisons between all intervention conditions will also be conducted to inform future implementation.

**Fig. 1:**
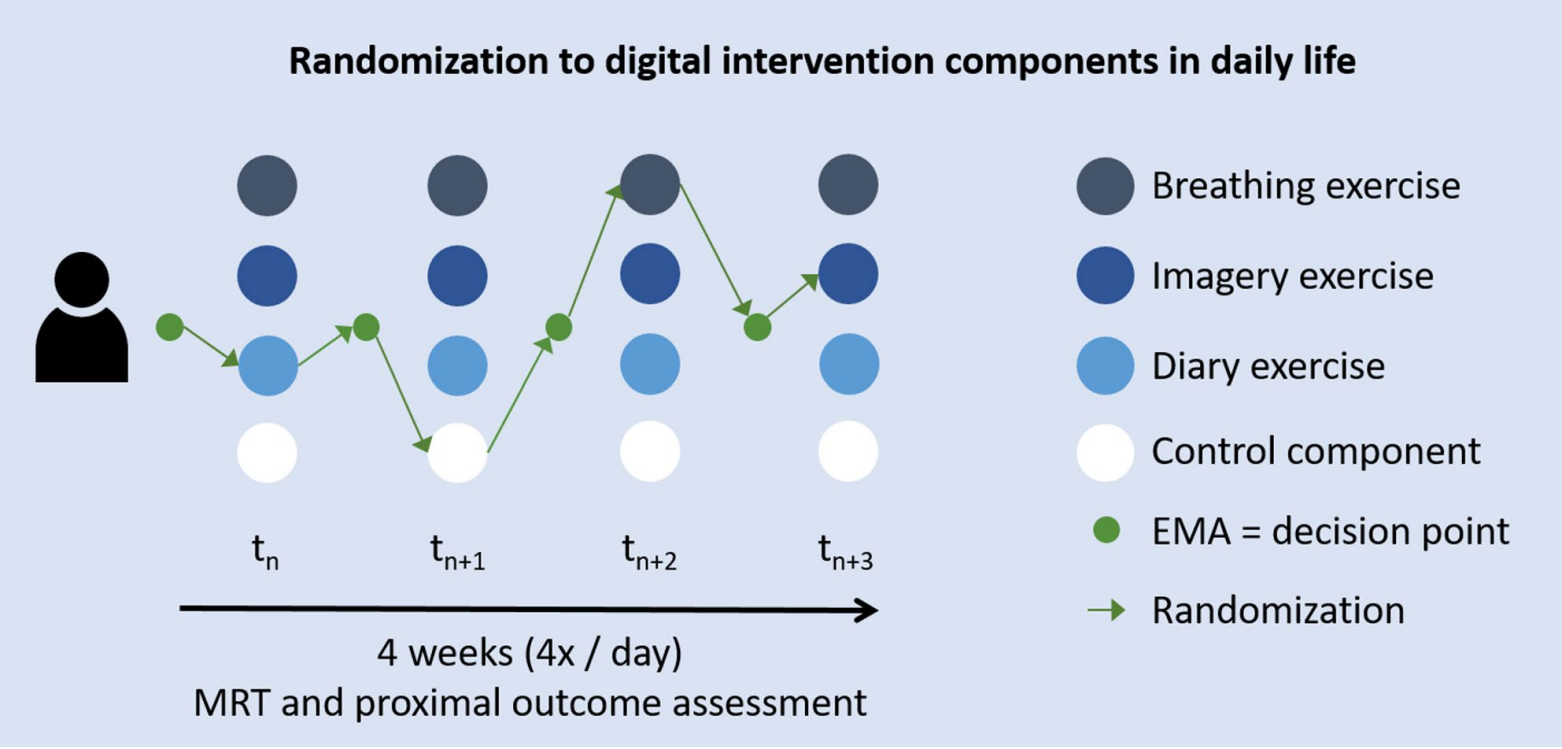
Micro-randomization of EMI components

Nested within the cluster RCT, we will conduct a micro-randomized trial [37] to determine the proximal effects of digital intervention components and what factors might influence those effects. Using repeated, within-participant randomization to all digital components of the MAISHA intervention, each participant in study arm A (digital and face-to-face elements) and B (digital only) will be randomized to digital intervention components (i.e., breathing, imagery, diary techniques, or control) at multiple time points (decision points) throughout the study. Decision points will be triggered in moments of high negative affect or stress, indicated by a participant’s EMA response crossing a pre-defined threshold (see Fig. 1).

### Participants

The study will recruit 666 young people aged 13–25 from Nairobi and the surrounding towns, Kenya, who exhibit difficulties in ER as assessed with the Difficulties in Emotion Regulation Scale (DERS) [38], i.e., an indicated population. Participants will be eligible if they score above pre-defined thresholds on the DERS-SF subscale, specifically: DERS Strategies = 3, Non-Acceptance = 3, Impulse = 2.5, Goals = 3.5, Awareness = 3.5, and Clarity = 3 [38, 39]. Young people experiencing acute suicidal thoughts or low functionality will be excluded but offered up to 2 counselling sessions (see Supplementary Table 1).

For a heterogeneous and generalizable sample, we will recruit young people from different socio-economic backgrounds, sexes, and educational levels. Recruitment will take place across at least four high schools, one university, and five community-based organizations (CBOs) or programs for out-of-school individuals in Nairobi to capture a broad range of social contexts. Recruitment logs will be closely monitored to ensure balance across these characteristics.

### Interventions

**Fig. 2:**
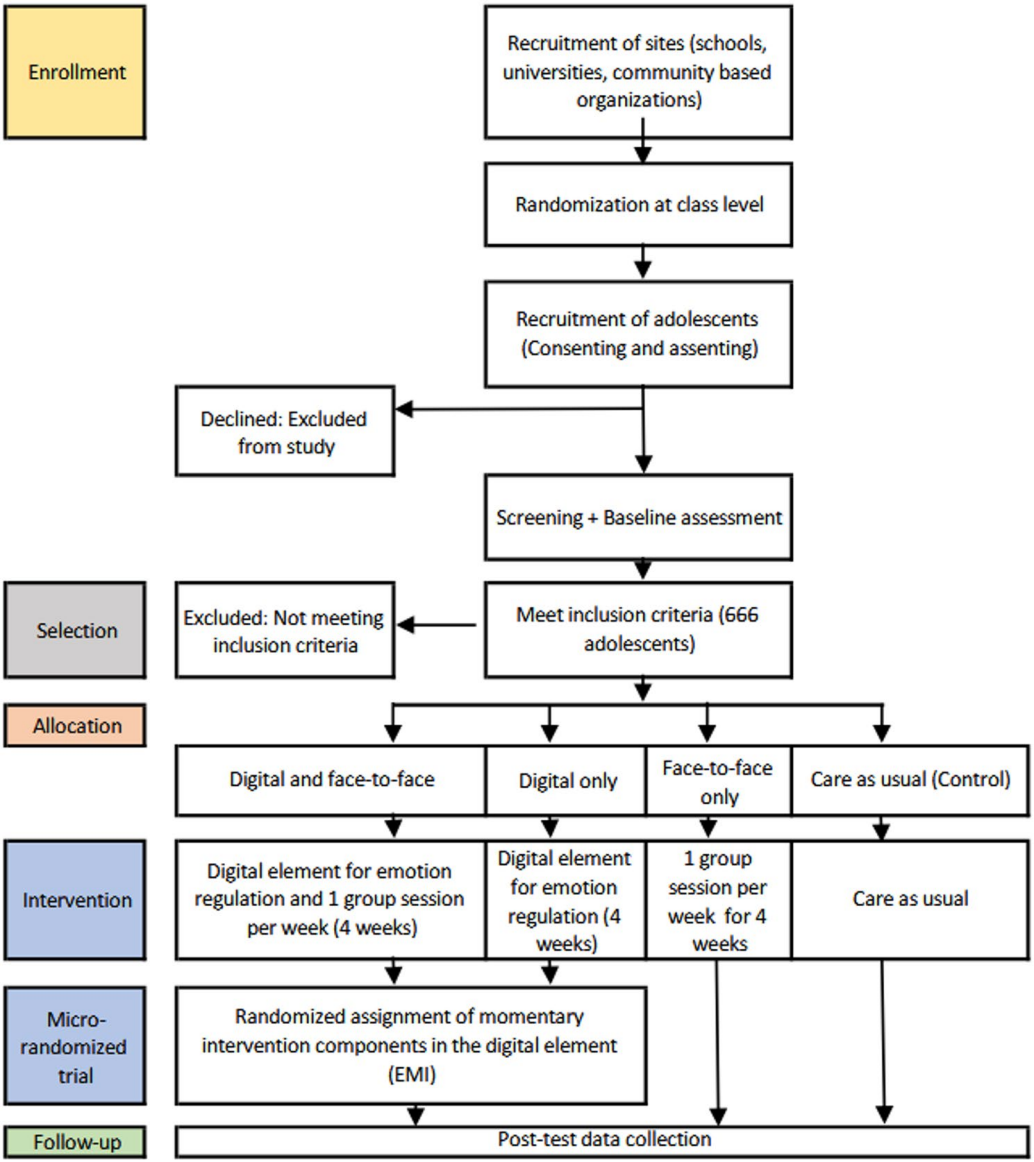
Study design and timeline

The blended intervention builds on existing digital and face-to-face elements, which were co-adapted following a human-centered design (HCD) process (see Fig. 2 and Supplementary methods 1). The co-creation and cultural adaptation of the blended intervention was done with twenty Kenyan young people aged 16–25 years with difficulties in ER – matching the trial’s target population (see participants section). Participants were involved in adapting the existing intervention via in-depth interviews, focus group discussions, co-design workshops, and a two-week feasibility test. The following section outlines intervention elements across the four trial arms: CAU, the digital element, the face-to-face element, and the blended intervention.

#### Control condition (Care as Usual)

CAU is broadly defined and includes standard health care services available within the public health system. This includes promotion, prevention, treatment, and rehabilitation of Mental, Neurological, and Substance Use (MNS) disorders for adolescents [40]. CAU is not actively monitored. All interventions will be given in addition to CAU. The control condition will also be put on a waiting list to receive an adjusted version of the digital element after the trial is finished.

#### Digital intervention element

The digital element of MAISHA is based on the AI4U training, originally developed to promote emotional resilience among young people in Germany [41]. AI4U itself builds on EMIcompass, a mobile EMI evaluated in an exploratory RCT among young people with early mental health problems [27]. The intervention will be delivered through MovisensXS (version 1.6.2 or higher), an mHealth application platform [42] that operates on Android smartphones. The app combines alarm-prompted EMA with EMI components, enabling real-time assessment and just-in-time delivery of ER exercises [26].

Participants will use their smartphone or a study phone to access the MAISHA app for 28 days. They will receive four EMA prompts per day at blocked-random (regular mood checks) and daily morning and evening check-ins. During the first 7 days, the learning phase, participants will be introduced to a new ER exercise per day (enhancing exercises) and reminded to practice previously learned techniques (consolidating exercises). In the subsequent 21-day training phase, participants will be prompted to do one consolidating exercise per day and apply the acquired skills in moments of high stress or negative affect, based on previous EMA ratings (interactive exercises).

Regular mood checks via EMA consist of two alternating item sets, each with 14 items covering affect and stress (see measures section). These data enable mood tracking and trigger the delivery of personalized EMI exercises to support emotional regulation in real-time. The EMI exercise content is informed by the compassion-focused therapy (CFT) approach [43] ^,^, operationalized in the app as three types of EMI exercises: breathing (e.g., count your breath), imagery (e.g., imagine a safe space), and diary techniques (e.g., success journal). In the micro-randomized trial, participants will randomly be assigned to one of the three exercise types or no exercise (i.e., control component) at each decision point. See Supplementary methods 2 for details on the digital intervention element.

#### Face-to-face intervention element

Building on the Kenyan Shamiri intervention [11], the face-to-face element of maisha involves four group sessions grounded in the wise interventions framework [44]: growth mindset (sessions 1 and 2), gratitude (session 3), and value affirmation (session 4). The sessions will run weekly for 1 h over four weeks, delivered in small groups of 6 to 7 young people by lay providers (aged 18 to 22). Supervision (ratio of 1:5) will be provided by trained clinical psychologists (see Supplementary methods 2).

#### Blended intervention (digital + face-to-face element)

**Fig. 3:**
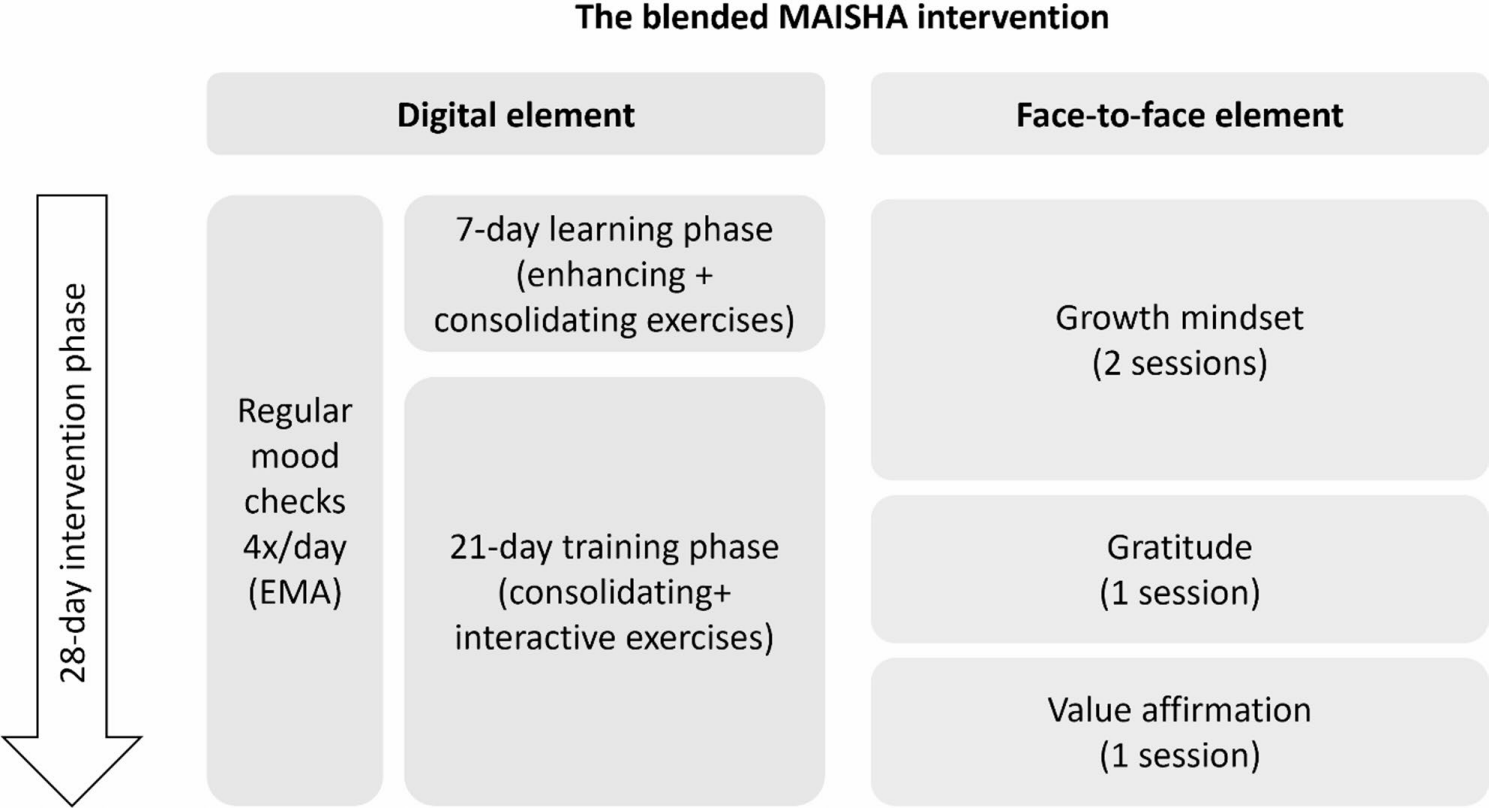
The blended MAISHA intervention

In the full blended intervention, both elements will be combined in a 28-day-long intervention phase in a parallel approach (Fig. 3). Fellows leading the face-to-face sessions will be trained to support participants with any potential issues in the digital element through a built-in chat.

### Outcome measures

#### Primary outcomes

The primary outcome will be ER difficulties at post-intervention, while controlling for baseline differences, assessed with the DERS-SF [38] (see Table 1). The DERS-SF is a validated self-report measure with 18 questions organized in six subscales and rated on a 5-point Likert scale. DERS-SF demonstrates good internal consistency (α = 0.89) [38] and acceptable test-retest reliability (*r* = 0.67) [45]. The total sum score across all subscales will be compared between the blended intervention and the control condition.

**Table 1:** An overview of measures and time points

Time points				
**Construct**	**Measures/Tools**	**Screening and Baseline (T0)**	**Throughout the intervention phase (T1)**	**Post-intervention (T2)**
*Screening measures*				
Sociodemographic characteristics	Demographic health survey questions on gender, age, education level, family economic status, and residence	X		
Functioning	Social and Occupational Functioning Assessment Scale (SOFAS) (63)	X		
Suicidality, Substance Use, Wellbeing	Community Quick Screening Tool (CQST) (66)	X		
*Primary outcome (Non-momentary)*				
Difficulties in Emotion Regulation	Difficulties in Emotion Regulation Scale (DERS-SF) (43)	X		X
*Secondary outcomes (Non-momentary)*				
Depression, anxiety, and stress	Depression Anxiety Stress Scale (DASS-21) (46)	X		X
Emotion Regulation	Emotion Beliefs Questionnaire (EBQ)(48)	X		X
	Emotion Regulation Questionnaire (ERQ)(49)	X		X
	Interpersonal Emotion Regulation Questionnaire (IERQ)(50)	X		X
Emotional reactivity	Short form of the Perth Emotional Reactivity Scale (PERS-S)(47)	X		X
Positive and negative affect	Positive And Negative Affect Schedule (PANAS)(45)	X		X
*Secondary outcomes (momentary)*				
Difficulties in Emotion Regulation	Eight alternating items of the State Difficulties in Emotion Regulation Scale (S-DERS)(56)		X	
Affect valence and arousal	Four bipolar VAS and one multiple-choice EMA item		X	
Event-related stress	Two VAS items assessing occurrence and intensity		X	
Emotion regulation capacity	Two alternating VAS items		X	
Social context	Two multiple-choice items		X	
Activities	One multiple-choice item		X	
Perceived Emotion regulation success (only Post-EMI EMA)	One VAS item		X	
Sleep quality (only morning check-in)	One VAS item		X	
Expectation about the day (only morning check-in)	One VAS item		X	
Reflection on the day (only evening check-in)	One VAS item		X	
*Process evaluation*,* Acceptability*,* Desirability*,* Usability*,* and Safety*				
Usability: digital element	MAUQ(59)			X
Acceptability: digital element	Unified Theory of Acceptance and Use of Technology (UTAUT)(67)			X
Acceptability and Desirability: digital and face-to-face element	Self-report questionnaire, In-depth interviews			X
Safety	Monitoring of SAEs		X	
YPAR experiences	Survey, reflective diaries, and in-depth interviews		X	X
Process evaluation: Trial delivery	Self-report questionnaire, attendance sheets, observation/audio ratings of selected sessions, in-depth interviews		X	X
Process evaluation: Implementation context	In-depth interviews			X
Process evaluation: Engagement	Self-report questionnaire, in-depth interviews, and FGDs			X

#### Secondary outcomes

##### Non-momentary outcomes

Non-momentary (i.e., assessed at baseline and post-intervention) secondary outcomes will include the level of positive and negative affect, symptoms of depression, anxiety, and stress, emotional reactivity, emotion beliefs, ER strategies, and interpersonal ER (see Table 1).

Positive and negative affect will be assessed with the Positive and Negative Affect Scale (PANAS) [46], a 20 item measure rated on a 5-point Likert scale. Total sum scores for negative and positive affect will be used for analysis. Symptoms of depression, anxiety, and stress will be measured using the 21-item Depression Anxiety Stress Scale (DASS-21) [47], with subscale scores (i.e., depression, anxiety, and stress) derived from 4-point Likert ratings. Emotional reactivity will be measured using the short form of the Perth Emotional Reactivity Scale (PERS-S) [48], an 18-item self-report measure rated on a 5-point Likert scale. Sum scores across the six subscales (i.e., activation, intensity, and duration for both positive and negative emotions) will be used for analysis.

Emotion beliefs (emotion controllability and usefulness) will be measured using the Emotion Beliefs Questionnaire (EBQ) [49], a 16-item self-report measure rated on a 7-point Likert scale. Sum scores across the four subscales (Negative Controllability, Positive Controllability, Negative Usefulness and Positive Usefulness) will be used for analysis. ER strategies will be assessed using the Emotion Regulation Questionnaire (ERQ) [50], a 10-item self-report measure rated on a 7-point Likert scale. Average scores for the two subscales (i.e., Cognitive Reappraisal and Expressive Suppression) will be used for analysis. The Interpersonal Emotion Regulation Questionnaire (IERQ) [51], a 20-item self-report measure rated on a 5-point Likert scale, will be used to measure interpersonal ER. Average scores for each subscale (i.e., Enhancing Positive Affect, Perspective Taking, Soothing, and Social Modeling) will be used for analysis. Additionally, average scores for all six subscales (i.e., Nonacceptance, Goals, Awareness, Impulse, Strategies, and Clarity) of the DERS-SF will be analyzed as secondary outcomes to investigate specific domains of ER in exploratory analyses.

##### Momentary (EMA) outcomes

Next to non-momentary outcomes, momentary outcomes (i.e., assessed multiple times per day) will be collected to examine the proximal effects of the digital intervention element within the micro-randomized trial [37] (see Table 1). Momentary outcomes will include negative and positive affect, event-related stress, stress reactivity, ER, and context.

The EMA items on affect, event-related stress, and social context will be based on widely used measures [52–54] that were recently harmonized in a Delphi process with more than 50 EMA experts from the German Center for Mental Health. Affect will be assessed using four two-ended items and a multiple-choice item with 13 affect facets. Event-related stress will be captured with two items assessing the presence and intensity of negative and positive events. Stress reactivity will be calculated as a within-person relation between stress and mean affect ratings [55]. Items for affect and stress will be the same in the alternating EMA sets.

ER difficulties will be measured using two alternating 4-item sets of an 8-item short version of the validated State Difficulties in Emotion Regulation Scale (S-DERS) [56], following recommendations from a validation in daily life [57]. The selected items in each alternating EMA set represent all four subscales: Non-acceptance, Modulate, Awareness, and Clarity. In addition, one item from the original AI4U evaluation [41] will be included in each EMA set to capture perceived ER capacity. Social context (two items in set A) and activities (one item in set B) will be assessed in an alternating way.

In addition to regular mood checks, momentary measures will include short EMAs after momentary interventions (post-EMI EMAs), morning and evening assessments. Post-EMI EMAs will encompass one affect item, one ER capacity item, and one item measuring perceived ER success. Morning and evening check-ins will include the same five affect items described above, as well as items to measure self-rated sleep quality, expectation, and reflection about the day, and fun activities (see Supplementary methods 3).

All EMA items will be rated on a visual analogue scale (VAS) of 0 to 100, unless otherwise specified. To supplement these ratings, an open-ended item will invite participants to describe their current issues, thoughts, or feelings.

##### Process evaluation

A mixed-methods process evaluation will be conducted using the framework developed by Grant et al. [58]. The process evaluation aims to understand the trial delivery, implementation context, and participant engagement. We will use purposive sampling to select participants from each intervention arm and across study sites to ensure that all subgroups are represented.

Under trial delivery, we will assess the reach, fidelity, dose, and adherence to the intervention. Reach will be measured using the proportion of eligible participants, the proportion that consented, drop-outs, and representativeness by demographic characteristics. Fidelity, dose of, and adherence to the face-to-face element will be assessed using a self-report questionnaire, attendance sheets, observation, and audio ratings of selected sessions, and in-depth interviews with lay providers (*n* = 10) and supervisors (*n* = 3). Fidelity, dose of, and adherence to the digital element will be assessed using error reports, app log files (e.g., number and duration of completed EMA and EMI enhancing, consolidating, and interactive exercises). Where feasible, participants who discontinue the intervention will also be invited to provide feedback.

For the implementation context, we will qualitatively assess the need for adaptation, barriers, enablers, and contextual factors (structural, environmental, and cultural) that may influence implementation and outcomes. For engagement, participants will fill in a feedback questionnaire about their experiences post-intervention. In-depth interviews (IDIs) and focus group discussions (FGDs) will be conducted with a purposive sample of young people (*n* = 20) from the three intervention arms.

##### Acceptability, desirability, usability, and safety

Acceptability and usability of the digital element will be assessed using the Extended Unified Theory of Acceptance and Use of Technology (UTAUT2) [59] and the 21-item mHealth App Usability Questionnaire (e.g., MAUQ) [60]. For the acceptability and desirability of the face-to-face and digital element, we will conduct in-depth interviews with 30 participants (10 from each intervention arm). We will assess safety through monitoring Serious Adverse Events (SAEs), defined as any untoward medical occurrence that results in death, is life-threatening, requires hospitalization, or results in significant disability. SAEs will be documented and reported to the ethics committees and regulatory bodies using standardized reporting forms and protocols [61]. Given the brief, guided nature of the psychosocial exercises and close facilitator supervision, the risk of SAEs is expected to be very low. Risks will be monitored through ongoing observation by trained facilitators and assessors, participant self-report, and brief check-ins after face-to-face sessions. Participants showing distress and those needing additional support will be referred to clinical psychologists or appropriate mental health services. Participation may be paused if continued involvement poses risk.

##### Youth participatory action research (YPAR)

To measure the participatory experience of youth researchers, we will employ a mixed-methods approach guided by the 7Ps Framework by Cahill and Dadvand (2018) [34]. This framework examines youth participation through seven interrelated dimensions: purpose, positioning, power relations, perspectives, protection, place, and process. Quantitative data will be collected using a structured survey with a 5-point Likert scale. We will collect qualitative data through weekly reflective diaries, monthly meetings, and exit in-depth interviews. The reflective diaries will be completed by the youth researchers with entries of planned activities for the week, achievements, personal reflections, challenges encountered, strategies used to address those challenges, and any support required.

##### Other measures

Other study parameters include pre-specified sociodemographic characteristics e, which will serve as equity indicators (i.e., gender, age, education level, family economic status, and residence) (see Table 1). These were chosen based on prior literature [62] and will be used for exploratory subgroup analyses to assess whether the effect of the intervention differs across subgroups [63].

### Procedures

#### Translation and cultural adaptation

Before the recruitment of participants, a WHO-guided adaptation process [64] was followed to translate and culturally adapt study materials to the Kenyan context. This included English versions of EMA items and digital intervention element content. The adaptation process included forward and backward translation with help from local experts and youth researchers, consensus team meetings, piloting tools for item clarity, and a final consensus meeting (Supplementary methods 4 and Table 2).

#### Screening and recruitment

The research team will contact schools, universities, and youth organizations to seek permission to engage with young people. Following initial visits and an official letter detailing the nature of the research, we will collaborate with the institution heads to organize the initial information sessions with young people. Interested young people will receive detailed study information by the research team, and before signing consent or assent (i.e., for minors) forms. Minors (i.e., below 18 years) will also get information sheets and consent forms for their guardians. Participation is voluntary, and young people can withdraw at any time. Data collected up to the point of withdrawal will be retained for analysis once anonymized.

After enrollment, screening will be used to identify who meets the inclusion criteria. Participants will fill in self-reported psychometric and sociodemographic measures (see above), and take a short Social and Occupational Functioning Assessment Scale (SOFAS) interview conducted by trained research staff [65].

#### Randomization and blinding

After screening and baseline data collection, participants will be randomized at the cluster level to one of the four study arms using permuted block randomization with blocks of four clusters each. To minimize the risk of cross-contamination within schools and small community-based organizations, naturally occurring sub-groups (e.g., class levels in schools) will be treated as allocation units and assigned to the same intervention arm. In case of community-based organizations, which are community-led groups based on shared interests (e.g., sports initiatives) [66], participants from a given CBO will be randomized to the same intervention condition. The randomization procedure will be carried out centrally and off-site using a computer-generated allocation sequence. Participant personal identification data will be stored separately from the process and outcome data. Assessors will be blind to the allocation of participants, data specific to the experimental groups will be stored separately and breaks in masking will be documented. Unblinding of assessors will only occur in exceptional cases where knowledge of the allocation is necessary for participant safety.

#### Data collection

Measures will be assessed at two time points: baseline (i.e., before randomization, also used for screening) and post-intervention (i.e., after the 28-day intervention period). To assess primary and secondary outcomes, participants will complete self-report measures using *Redcap* [67], a secure electronic data capture system with built-in quality control features (e.g., logic checks), and participate in interviews and FGDs (see Table 1 - Additional File 2). All data collection tools will be administered in English.

During the intervention phase, participants assigned to the digital or blended intervention arms will receive four mood checks daily with two alternating EMA item sets each day, scheduled at random within set blocks of time. For proximal effects assessment, participants will be prompted to reply to a post-EMI EMA with three items after each momentary intervention component. Morning and evening EMA will be collected at participant-defined times. A research team member will routinely review incoming data for completeness and consistency. Additional data cleaning steps will be performed before analysis to ensure data integrity.

Qualitative data collection will follow the principle of information power, i.e., stopping at the point at which no new information is obtained and redundancy is reached [68]. If possible, interviews will be conducted face-to-face.

Data collection will be conducted by a research team consisting of a PI, co-investigators, doctoral students, youth researchers, and local researchers. The data collection team will be fluent in English with experience in using both quantitative and qualitative methodologies.

The feasibility of all tools, including EMA, was piloted in the co-design phase with adolescents and young people who tested the questionnaires, EMA, and EMI over 2 weeks. Their feedback indicated that the number and frequency of items were manageable. Based on this pilot, several adaptations were implemented to further reduce participant burden, such as allowing users to adjust prompt times and enabling a ‘do-not-disturb’ mode.

### Data processing and analysis

#### Quantitative data

A detailed statistical analysis plan will be published before conducting any analyses. Data will be accessed only after completion of the final post-intervention data collection. Data will be analyzed according to the intention-to-treat principle. Data processing will involve cleaning, (re-) coding, and formatting of variables for statistical analysis. The sample size was calculated using the Shiny CRT Calculator [69]. Using a parallel design with baseline measure and assuming an average intra-cluster correlation (ICC) of 0.05 [70], and α = 0.05, 25 clusters per arm with 6 participants each (600 participants) will yield 80% power (β = 0.8) to detect a mean difference of ≥ 0.36 between the blended intervention and the control condition (CAU). The assumed minimal mean difference aligns with effects observed in previous RCTs evaluating the individual intervention elements [27, 30]. Considering a 10% dropout rate, 666 young people will need to be recruited.

The primary analysis will apply a mixed-effects linear regression model to examine the effect of the blended intervention compared to the control condition (CAU) on emotion regulation difficulties. The level of ER difficulties will be compared between the experimental and the control conditions at post-intervention. ER difficulties (DERS-SF) at post-intervention will be entered as the dependent variable, and ER difficulties at baseline, condition (= allocation), and setting (= location or context in which participants receive the intervention) will be entered as independent variables. While random allocation to intervention arms is designed to balance baseline characteristics, we will verify balance post-randomization and – if imbalance is identified – incorporate appropriate covariate adjustments to improve precision and validity of effect estimates. To account for clustering, the model will include a random intercept for the allocation unit (i.e., face-to-face group), with individual (level 1) nested within these units (level 2), reflecting the two-level structure. The model will be fitted using Restricted Maximum Likelihood (REML), and the main effect of condition will be estimated using a Wald-type test (α = 0.05). Potential regression to the mean will be considered in the interpretation of results.

Secondary measures - including positive and negative affect, symptoms of depression, anxiety, and stress, emotional reactivity, emotion beliefs, ER strategies, and interpersonal ER - will be analyzed using mixed-effects linear regression models, consistent with the approach described for the primary analysis. Exploratory analyses comparing the other intervention conditions (e.g., face-to-face and digital only against CAU) will be conducted to explore the potential contribution of intervention elements to overall effects and generate hypotheses for future implementation.

Exploratory analyses will include subgroup analysis to examine whether signals of efficacy of the blended intervention differ across specific groups. These analyses will be conducted within the primary mixed-effects model using interaction terms. We will examine proximal signals of efficacy of digital intervention components using data from the micro-randomized trial. Mixed-effects linear regression models with EMA observations (level 1) nested within individuals (level 2) will be applied to account for the hierarchical structure of longitudinal EMA data. Variables will include momentary negative affect, stress, and momentary ER. EMA data before and after momentary intervention components will be compared and types of EMI exercises (i.e., breathing, imagery, diary, control) will be included as independent variables. Results from all exploratory analyses will be interpreted using effect sizes and 95% confidence intervals rather than formal significance testing. Data from the process evaluation, acceptability, desirability, usability, and safety, sample characteristics, and baseline measures will be summarized descriptively.

We assume data will be Missing at Random (MAR) and will explore missingness patterns to assess this assumption. Continuous variables will be presented using means and standard deviations or medians and interquartile ranges; categorical variables using frequencies and percentages. All statistical analyses will be conducted in R.

#### Qualitative data

We will take detailed notes and audio records during interviews and FGDs. Audio recordings will be transcribed verbatim and with a secure, AI-based transcription software, e.g., Vink [71]. To maintain confidentiality, we will conduct interviews in a secure space, not record any personal identifiers alongside the transcripts, and anonymize transcripts before analysis. We will conduct a codebook thematic analysis [72] to identify patterns, themes, and meanings within the data, combining inductive coding with framework-informed interpretation [73] Routine debriefings during data collection will inform the draft codebook [74]. Consistency and rigour will be ensured through collaborative coding and iterative refinement in regular team discussions. A sample of transcripts will be used to detail the initial codes before applying them to the whole dataset using NVivo [75] or a similar software.

## Discussion

MAISHA is an innovative, culturally adapted, blended intervention aimed at improving ER in young people. This cluster-randomized 2 × 2 factorial trial evaluates its efficacy in reducing ER difficulties (primary outcome) compared to care as usual (CAU) in Nairobi, Kenya. Co-designed with young people, the intervention is based on a face-to-face group element led by lay providers and a digital ecological momentary intervention (EMI) via an mHealth app. Both elements have shown signals of efficacy in reducing emotional reactivity and internalizing symptoms [27, 30].

Growing evidence highlights the potential of blended ER interventions in youth and adolescent mental health. Meta-analyses indicate moderate effects on ER difficulties [76] and a moderate to large effect on internalizing symptoms [77], supporting their use in both clinical and preventive contexts. Blended interventions also address common limitations of purely digital interventions, including technical barriers, lack of personalization, and high attrition rate [78, 79], while promoting real-world relevance. The EMI approach specifically supports transfer of ER skills by delivering just-in-time support [80] during emotional challenges in daily life, contextually targeting ER as a key mechanism of mental health.

Accordingly, we hypothesize that young people receiving the full blended MAISHA intervention will demonstrate less ER difficulties post-intervention, along with improvements in secondary outcomes (e.g., depressive and anxiety symptoms), compared to those receiving CAU.

### Strengths and limitations

This study has several notable strengths. First, it employs a rigorous RCT design, including a CAU control group, and adheres to CONSORT and SPIRIT guidelines for trial reporting [36]. Second, while not powered for secondary analyses, the 2 × 2 factorial design enables exploratory comparisons between individual intervention elements, offering initial insights into their contributions to overall effects. Third, designing the digital element as an EMI helps to target ER in daily life, where it is most relevant. Fourth, the intervention was culturally adapted with young people through a human-centered design approach, and youth researchers will be involved to ensure it remains grounded in their lived experiences and needs. Fifth, this is one of the few trials to evaluate a blended ER intervention in an LMIC setting, addressing a critical gap in youth mental health and scalability research.

Nevertheless, the study has some limitations. First, it lacks an active control group but compares the intervention to a broadly defined CAU. We chose this comparison to reflect real-world conditions and partially mitigated this weakness by undertaking a 2 × 2 factorial design. Second, participants are not blinded, which may introduce a Hawthorne effect [81]. Blinded assessors remain the most pragmatic strategy for this trial, as for most psychosocial interventions. Third, peer interaction across groups may lead to contamination. We reduce this risk by clustering participants within the same class level.

### Implications for practice

Transition from childhood to adulthood can be an emotionally stressful time, and the need for support in ER is high. Blended interventions that strengthen ER and well-being may help address this gap. The MAISHA intervention has the potential to improve young people’s ER, thereby lowering the risk of developing mental health conditions later in life. In addition to testing intervention effects, this study will generate methodological insights for the design of future cultural adaptation studies, supporting scalability and context-appropriateness of mental health programs in LMICs. It will also contribute to the growing evidence base on mechanism-informed interventions, particularly EMIs [82–84]. If MAISHA demonstrates efficacy in improving ER difficulties, it may provide a scalable approach for improving young people’s mental health in other regions of Kenya and beyond. Future implementation-focused trials could test its effectiveness under real-world conditions and explore its transferability across settings.

## Supplementary Information

Supplementary Material 1.

## Data Availability

We will make data and materials available wherever possible on formal request. All data collected in the context of this study will be managed at the Heidelberg Institute of Global Health. Data sharing will take place in compliance with the data sharing policy and established procedures. Third-party researchers who are interested in the data must complete and sign data sharing agreements. Decisions on data sharing will be made on a case-by-case basis by the investigators upon reasonable request. Individuals granted database access will be expected to use the data to answer the questions clearly outlined in the data sharing agreement.

## Abbreviations

CAU: Care as usual
CBOs: Community-based organizations
CFT: Compassion-focused therapy
CQST: Community Quick screening tool
DASS-21: Depression Anxiety Stress Scale
DERS: Difficulties in Emotion Regulation Scale
DERS-SF: Difficulties in Emotion Regulation Scale - Short Form
EBQ: Emotion Beliefs Questionnaire
EMA: Ecological momentary assessment
EMI: Ecological momentary interventions
ER: Emotion Regulation
ERQ: Emotion Regulation Questionnaire
FGD: Focus group discussion
HCD: Human-centered design
LMICs: Low- and middle-income countries
IERQ: Interpersonal Emotion Regulation Questionnaire
MAR: Missing at Random
MAUQ: mHealth App Usability Questionnaire
MRT: Micro-randomized trial
RCT: Randomized controlled trial
SSA: sub-Saharan Africa
PANAS: Positive and Negative Affect Scale
PERS-S: Perth Emotional Reactivity Scale
SAEs: Serious Adverse Events
SOFAS: Social Functioning Assessment Scale
UTAUT2: Extended Unified Theory of Acceptance and Use of Technology
YPAR: Youth participatory action research
