# Supplementary material for: Efficacy and acceptability of a blended intervention for emotion regulation (MAISHA) among young people in Kenya: study protocol for a cluster RCT
Source: BMC Psychol. 2025 Dec 20;14:114. doi: 10.1186/s40359-025-03837-z (PMC12837527; doi:10.1186/s40359-025-03837-z)
Supplement: Supplementary file 1 — Supplementary Material 1. [file 40359_2025_3837_MOESM1_ESM.docx]

**Supplementary Material**

Esther Rugendo^1,2,†^, Glorialoveness Lyimo^3,†^, Rahim Daya^2^, Moreen Githinji^2^, Kevin Kashamba^4^, Faith Njoki^2^, Innocent Yusufu^4^, Tom Osborn^2^, Mary Sando^4^, Azan Nyundo^5^, Till Bärnighausen^1^, Ulrich Reininghaus^5,6,7^, Shannon A. McMahon^1,‡^, and Annika Stefanie Reinhold^5,7,‡^

Supplementary Table 1. Eligibility criteria

Supplementary methods 1. Co-Design and cultural adaptation of the intervention

Supplementary methods 2. Intervention description

Supplementary methods 3. Momentary measures

Supplementary methods 4. Adaptation of tools to other languages

Supplementary Table 2. Tools adaptation

Supplementary methods 5. SPIRIT 2025 checklist

References

**Supplementary Table 1. Eligibility criteria**

| **Inclusion Criteria** | **Exclusion Criteria** |
| --- | --- |
| - Age range: 13-25 years - Fulfillment of criteria for early symptoms of emotional dysregulation, that is: participants exhibit difficulties with emotion regulation (screening with DERS-SF above the defined threshold, i.e, DERS Strategies = 3, Non-Acceptance = 3, Impulse = 2.5, Goals = 3.5, Awareness = 3.5, and Clarity = 3). These cut-off scores for DERS-SF subscales are informed by studies with similar populations [1,2] as well as expert opinions. - Voluntary enrollment into the study (written consent) | - Individuals with low functionality (i.e., due to severe mental illness such as an acute psychotic episode); assessed with the SOFAS, we excluded individuals with a total score below 50, following guidance from the Clinical High at Risk Mental State (CHARMS) study [3]. - Persons with severe physical illness that impedes the use of intervention (i.e, Severe physical disability like total blindness, severe motor impairments) - Current suicidal ideation, assessed with the Community Quick Screening Tool (CQST). This includes two specific items: “In the past, have you ever attempted suicide? rated on a scale 0=No, 2 = Don't know/No answer” and “Over the past 2 weeks, has the thought of harming yourself occurred to you? rated on a scale of 1=Yes, 2=No. - Consumption of alcohol or other psychotropic substances on the day of the assessment (self-report, CQST). This will be assessed using two questions: First, “How often do you take drugs/a drink containing alcohol?” which will be rated on a scale from 0= Not at all 4= 4 or more times a week. Second, “How many drugs do you use or alcoholic drinks do you take on a typical day when you take drugs or drink? rated on a scale 0=1 or 2, 4 = 10 or more - Evidence that symptoms of psychological distress are precipitated by an organic disease, i.e. epilepsy, brain tumor, or other significant neurological diseases - Insufficient command of the local language (i.e., English or Swahili) - Participants or guardians refusing or withdrawing informed assent or consent |

*NOTE*: Suicidal ideation will be assessed with two questions (mentioned above). If a participant responds "YES" to either question, they will be referred to the supervisor, in this case, a clinical psychologist, for further risk assessment. Based on this evaluation, the psychologist will determine the participant’s eligibility for inclusion in the study. Participants assessed as being at no acute risk may proceed with the study. However, if they are found to be at significant risk, they will not be included in the study and will instead continue receiving counselling support from the clinical psychologist. If necessary, they will be referred to appropriate mental health services for further care.

**Supplementary methods 1. Co-Design and cultural adaptation of the intervention**

Human-centered design (HCD) research, which involves co-creating, testing, and culturally adapting interventions, can produce compelling, accessible, and acceptable interventions [4,5]. HCD is a flexible, participatory, iterative approach to creating solutions by putting people at the center of the process [6]. The HCD process typically begins with developing a deep understanding of the needs and experience of the end users (Empathize), followed by defining the core problem (Define). The process continues with brainstorming a range of creative ideas (Ideate), building a representation of ideas (Prototype), and evaluating them through user feedback and testing (Test). This process ensures that solutions are contextually relevant [7]. We conducted the HCD process in two countries in East Africa: Kenya and Tanzania. Even though the intervention tested in this trial is based solely on the version developed through the Kenyan process, we wish to acknowledge the valuable contributions from participants in Tanzania. Their input is helping to generate early insights into the potential transferability and contextual adaptation of the intervention to other settings. Below is the summary of HCD activities conducted in each country.

First, we conducted a kick-off workshop with young people who met the same inclusion/exclusion criteria as the ones for the trial. This kick-off workshop aimed to gather perspectives on youth mental health, emotional regulation, and explore resources available to them for emotion regulation. In addition, we conducted a kick-off workshop with stakeholders (n in Kenya = 19), including parents or guardians of young people, fellows, supervisors, the clinical psychologist, a representative from youth-based organizations, and teachers. The kick-off workshops were used as a basis to create short storyboards on typical scenarios in which young people face strong emotions. A graphic artist supported the development of three storyboards in each country, which were used in subsequent Focus Group Discussions (FGDs).

The FGDs involved the same young people in the kick-off workshop. We conducted three FGDs in each country, each having 6-8 participants. Discussions focused on difficulties that young people face when dealing with strong emotions, how they cope with emotions in daily life, and where they might see a need for support. During the FGDs, we conducted a mix of a) “playful” getting-to-know activities, b) silent brainstorming, c) discussion in the larger group, and d) discussion about three concrete emotion regulation scenarios supported by the three visual storyboards.

Subsequently, we carried out in-depth interviews in each country (n in Kenya = 18) to gain deeper insight into emotion regulation situations and mechanisms in daily life. Following this, we carried out a transferability test where the same young people (n in Kenya = 18) used an initial version of the blended intervention (digital and face-to-face elements) in their daily lives for two weeks. The 2-week transferability test is a short version of the blended intervention. In week one, participants completed the learning phase of the digital element and face-to-face session, followed by a training phase and the second face-to-face session in week 2. During the transferability test, quantitative data were collected, including EMA ratings, compliance with the digital and face-to-face components, and other usage behavior measures (i.e., length of interaction) to inform further adaptation of the intervention components.

For a deeper understanding, we sought qualitative feedback from participants through in-depth interviews. In-depth interviews were conducted in each country (n in Kenya = 11) to understand user experiences with the digital element and face-to-face sessions, challenges, and recommendations for improvement.

Building on insights from the transferability test of the initial intervention and post-test in-depth interviews, we conducted an interactive co-design workshop with participants (n in Kenya = 18) to further adapt the intervention. During this workshop, young people worked in small groups of 2-4 to optimize different components of the intervention and its delivery, coming up with visual prototypes. Co-creation considers the design and content, including wording, pictures, audio recordings, text, alarm/mood checks, and support processes.

Thereafter, we conducted in-depth interviews with participants (n in Kenya = 18) to gain a perspective of their experiences with the HCD process and seek their opinions on Youth Participatory Action Research (YPAR). To support the continued adaptation of the intervention components beyond the co-creation workshop, we engaged two youth researchers in each country. Importantly, these youth researchers were not selected from among the participants involved in the HCD process but were independently recruited. The HCD process was further guided by the cultural adaptation framework suggested by Heim and Kohrt [8]. This entails assessing cultural concepts of distress and emotion regulation, intervention component transfer, and intervention delivery.

**Supplementary methods 2. Intervention description**

**The digital intervention element**

The MAISHA digital intervention element draws upon the concept of Ecological Momentary Intervention (EMI) [7]. EMIs build on Ecological Momentary Assessments (EMAs), which utilize repeated real-time assessments (i.e., mood checks) in daily life situations to collect dynamic time-series data on moods and behavior [9]. These data are then harnessed for an EMI to offer momentary interventions that can target momentary mechanisms (i.e., emotion regulation) in real-time and the real world.

The EMI is delivered by an app built on the MovisensXS platform and operating on Android smartphones. It functions in two phases: the learning phase and the training phase. During the learning phase (7 days), participants will receive daily EMA prompts, be introduced to a new enhancing exercise each day, and be prompted to practice their skills with consolidating exercises twice per day. This is followed by a 21-day training phase. Next to daily EMA prompts, this phase includes one consolidating exercise per day, which encourages continued practice of previously introduced exercises as well as interactive exercises designed to support real-time application of these skills. Further, exercises will be available to participants on their demand.

In both phases, the app prompts users four times a day at random within set blocks of time to fill out an EMA (i.e., mood check). Participants will be able to set their preferred time frames to receive the mood checks. In addition, participants will receive a morning and an evening check-in with a different set of questions at a self-defined time point. To maximize compliance and increase the probability of engaging with the exercises, participants a) can postpone each prompt up to 5, 10 minutes, b) are offered two different sets of EMA questions for a higher variety, c) receive gamification points for answering to prompts. Mood checks allow participants to track their emotions by answering brief questions about their current affect, stress, emotion regulation, and context. Additionally, these regular check-ins are the foundation for the delivery of timely, personalized intervention components to support emotional regulation. Specifically, the EMA will trigger a momentary intervention when participants report negative affect (i.e., they feel bad, unsatisfied, or unwell with a score < 42) or a negative event (rate with intensity of > 58). These thresholds have been predefined based on evidence from prior studies,[10] ensuring support is delivered when participants are most likely to benefit.

For this, three types of intervention components are provided and compared to a control component: Breathing, imagery, and diary exercises. All exercises are informed by a compassion-focused therapy (CFT) approach. CFT integrates Cognitive Behavioral Therapy with insights from evolutionary psychology, attachment theory, and mentality theory, and aims at helping individuals address self-criticism and shame by cultivating compassion and acceptance for themselves and others. By targeting an overactive threat system, CFT techniques have been shown to reduce negative affect and emotional reactivity in laboratory studies [11] and in daily life [10] .

The breathing exercises are designed to help participants feel calm and focused. Each exercise lasts 2-3 minutes, depending on the delivery scheme (i.e., interactive exercises are shorter than enhancing and consolidating ones). In *Counting your breaths,* participants are instructed to find a comfortable posture, either sitting, standing, or lying down, and take a few slow, deep breaths. The exercise involves silently counting each inhale and exhale up to five, with a short pause in between breaths. *Breathing with pauses* is a guided breathing exercise with intentional pauses to encourage mindfulness and relaxation. It involves slow, conscious inhaling, brief holding of the breath, and exhaling with a pause.

Imagination exercises include my calm and safe space, a compassionate companion, and emotions as waves*. My calm and safe place* encourages participants to imagine a place that feels calm and safe to them. *A compassionate companion* encourages the imagination of a supportive figure, real or imagined, human or not, that shows unconditional kindness, understanding, and acceptance towards the participant. They then recall an unpleasant situation and invite their compassionate companion to help soothe any distress that arises. *Emotions as waves* guides the participant to imagine their emotions as natural, flowing waves and learn to stay present with their emotions instead of being overwhelmed or trying to suppress them.

Diary exercises are widely implemented interventions aimed at supporting wellbeing and emotional awareness. Studies demonstrate that even brief structured activities can lead to improvement in mood and resilience [12,13]*. The diary of successes* encourages participants to reflect on and record small achievements or positive moments in real-time, such as situations in which they feel competent, proud, or satisfied with their actions. The *diary of joyful moments* encourages participants to reflect on and capture positive experiences in real time. Participants note moments that brought them joy, comfort, or satisfaction.

The control component has no intervention. Instead, participants will be notified to fill out the post-intervention assessment after a short delay of up to one minute. Participants will use their smartphones to access the digital intervention element or be provided with a study phone, configured with restricted access to other apps where possible. Those without a compatible device will be provided with a study phone, configured with restricted access to other apps, for the duration of the intervention. Study staff and fellows will support participants to set up the app and guide them to select a time frame that works best for them to use it, and can be contacted by participants for basic support (e.g., on technical issues) through a built-in chat option. The app operates offline with local data storage, which is uploaded to a secure server when internet access is available.

**The face-to-face intervention element**

The face-to-face intervention is adapted from the Shamiri Intervention [14], a locally developed group format rooted in a tiered caregiving model and provided by lay providers. Its content is grounded in the WISE interventions framework, which employs brief evidence-based strategies that focus on key positive psychological processes such as belonging, self-efficacy and adaptive meaning-making rather than psychopathology or deficit-based models [15]. The face-to-face element will include small group sessions where participants learn more about three main topics: growth mindset, gratitude and value affirmation. The sessions will run for four weeks, with one session each week lasting for one hour. In the first two sessions, participants will learn about the *growth mindset.* In week 1, group leaders will explain the concept of growth, personal development, and neuroplasticity (i.e., the ability of the brain to grow and people’s ability to learn and improve with practice and effort). The participants will write stories about their personal growth and about how they could use growth to solve specific problems. In the second week, participants will discuss strategies for overcoming life challenges using a framework for problem solving, and write a letter to a friend to explain what they have learned, and how they are applying problem-solving skills to a specific life challenge.

In the third session, participants will discuss the importance of gratitude, write a gratitude letter to someone, and list 3 things for which they are grateful on each day of the following week. In the fourth session, participants will learn about values, and select the values that are important to them from a list provided. They then write about a time when they demonstrated one of the values, reflect, and write a plan on how they might practice one of their values in the future. In this last session, participants will also reflect on the most helpful lesson from the sessions and how they can continue applying their skills in the future.

During the RCT, all four sessions will be delivered in 4 weeks. The intervention will be delivered in small groups of 6 to 7 students, led by trained lay providers (i.e., fellows), who are young adults (aged 18 to 22) from the local community. The fellows are trained for 2 days on the contents of the intervention (both digital and face to face), small group facilitation, counseling techniques, and safety protocols. During the first session the face-to-face intervention, participants will receive training on how to use the MAISHA application facilitated by the fellows with support from a member of the research team.

**Supplementary methods 3. Momentary Measures**

**Momentary (EMA) measures.**

Participants will be prompted 4 times a day at block randomized times to answer EMA questions on affect and stress (i.e., mood check). Regular mood checks will be offered by the app, which will be used to personalize an interactive task in moments of high stress.

**The EMA items** are designed to assess affect, event-related stress, emotional regulation, and context. Items are based on established measures [16–18] that were recently harmonized in a Delphi process at the German Center for Mental Health [19] *Affect valence and arousal* will be measured using the following four bipolar items on a visual analogue scale (0-100): “At the moment I feel … bad–good / unsatisfied–satisfied / unwell–well / tired–awake”. In addition, a multiple choice item will be used to assess thirteen affect facets (i.e., sad; angry; anxious; lonely; nervous; stressed; active; happy; confident; relaxed; down; cheerful; ashamed). *Event-related stress* will be measured on visual analogue scale (0-100) through two item assessing both the occurrence and intensity of recent events: “Have you experienced one or more negative events? How intense was the most significant one? (“not at all” if you haven't experienced a negative event)”; “Have you experienced one or more positive events? How intense was the most significant one? (“not at all” if you haven't experienced a positive event)”. *Emotional regulation difficulties* will be assessed with a visual analogue scale (0-100, not at all – very much) using the following alternating items. EMA Set A: “I am paying attention to how I feel”, “I have no idea how I am feeling”, “I am paying attention to how I feel”, “I am irritated with myself for feeling this way”, “My emotions feel out of control”, “I'm acknowledging my emotions right now”. *Emotional regulation capacity* will be measured by two alternating items on a visual analogue scale (0-100, not at all – very much). Set A: “I can change my feelings into the positive”; Set B: “I can cope with any difficulties I might face”. In the EMA Set A, *social context* will be measured with 2 items: “Who am I with? … alone; with others; with others online/on the phone” and “I am with … partner; family/relatives; friends; colleagues/classmates/ fellow students; acquaintances; strangers; care providers; others”. EMA Set B assesses *activities* using the item: “What am I doing right now? … Work, school, university; Resting, relaxing; Everyday tasks, shopping; Inactive leisure activities (reading, browsing the internet, social media, games, watching TV); Active leisure activities (sports, hiking, playing); On the move (on foot, car, public transport); Having a conversation; Self-care, eating, drinking; Caring for others (children, relatives, partner); Housework; Nothing; Other”. In addition, participants are invited to respond to two alternating *open text items*: “These issues, thoughts, or feelings occupy me the most at the moment” (Set A); “What's taking up most of your headspace at the moment?” (Set B)

**Post-EMI EMAs** include one bipolar item measuring *affect* (“At the moment I feel … bad–good”), one item measuring *ER capacity* (“I can cope with any difficulties I might face”, and one item measuring *perceived ER success* (“How successful were you at managing your emotions the way you intended to?”). All items will be rated on a visual analogue scale (0-100)

Participants will also receive **a morning and evening check-in** at self-defined times**.** In addition to the five items assessing *affect* (see above) the **morning check-in** measures *self-rated sleep quality* (“I slept well tonight”, and *expectation about the day* (“At the moment, I'm looking forward to the day” on a visual analogue scale (0-100). Moreover, participants will be asked whether they want to *plan a fun activity* for the day. If they respond with “Yes”, they can choose specific activities from various categories including a) Contacts & Socializing, b) Hobbies & Leisure c) Sports & exercise, d) Culture & Education, e) Nature & Garden f) Treat & Self-Care. Participants may also enter a custom activity of their choice if it is not listed under the predefined categories.

**The evening check-in** will include the five *affect* items described above, and one item to *reflect on the day*, rated on a visual analogue scale (0-100) (“Looking back, I feel positive about how today went”), and one item on their *fun activity* (“Were you able to do the fun activity that you planned for today? … yes / no / I did not plan one”).

Each completed response will be automatically time-stamped by the application. Data will be assessed, uploaded, and stored on both the device and MovisensXS servers.

**Supplementary methods 4. Adaptation of tools to other languages**

The study implemented a cross-cultural adaptation process [20] to translate and modify existing measures for relevance in the East African context. This process was informed by and adapted from a WHO guideline on the adaptation of psychological interventions [21][21]Tools originally developed in German were first translated into English for use in Kenya, where this RCT will be conducted. To support broader contextualization and as part of the co-design process (see eMethods 1), all measures were subsequently translated from English into Swahili for use in Tanzania.

The adaptation process involved using forward and backward translation, committee review, and pre-testing. The full adaptation process involved the following seven steps.

1. **Team formation:** A team of three bilingual reviewers and/or cultural experts, including one MAISHA research team member, a local mental health expert, and a local representative, was formed.
2. **Independent forward translations**: Two bilingual reviewers/cultural experts independently did the forward translation of the MAISHA project tools for use in the target population. This included literal translation of individual words and sentences from one language to another and an adaptation with regard to idioms and to cultural contexts.
3. **Consensus team meeting (resulting in Consensus Draft 1)**: The forward translation team reviewed the translated documents together to identify and propose resolutions for discrepancies before the backward translation.
4. **Independent backward translation:** A third bilingual reviewer/cultural expert (with no exposure to the original content) independently translated items generated from forward translation back into the original language
5. **Second consensus team meeting (resulting in Consensus Draft 2):** The three linguistic/cultural experts (the two doing the forward and one doing backward translation) had a consensus meeting to read and document the discrepancies between the forward and backwards translations. Accuracy of the items or equivalence to the original content, acceptability in the target population, and any risk for eliciting biases were discussed. The team then discussed discrepancies and problematic items and decided upon an outcome. A master document was then prepared for piloting.
6. **Pilot item clarity:** The adaptation team conducted a pilot exercise to collect data regarding the clarity of translated items. Qualitative feedback was collected from a small sample (n = 2-5) of people from the target population in the respective country.
7. **Final team meeting (resulting in Consensus Final Draft):** Each member of the adaptation team independently reviewed the master document from steps 5 and 6, provided feedback on items that were unclear, problematic, or potentially unnecessary, and suggested alternative items where appropriate. The team then discussed these inputs and reached consensus on the final draft.

Some of the tools were adapted using a shortened process, which excluded steps 4 and 5 for feasibility reasons. The decision on which tools should be adapted in the long or short process was based on the following considerations: 1) the linguistic dynamism of the tools (wording accuracy required). A high accuracy is required for all outcome measures, 2) the number of pages showing the approximate effort and resources required, and 3) the ability to adjust content mid-course (need for flexibility and revision based on insights gathered during the research process, as is common for qualitative measures, e.g., interview guides).

The table below outlines the specific tools translated, the source languages, and the adaptation steps applied to each tool.

**Supplementary Table 2: Tools adaptation**

| **Tools** | **Original and validation language** | **Target language** | **Context** | **Forward (Step 1)** | **Back-ward (Step 4 + 5)** | **Small pilot (Step 6)** | **Final consensus meeting (Step 7)** |
| --- | --- | --- | --- | --- | --- | --- | --- |
| 1. Screening / Global outcome measures | English | Swahili | Kenya & Tanzania | Double + consensus | Yes | Yes | Yes |
| 2. Momentary measures | German | English and Swahili | Kenya & Tanzania | Double + consensus | Yes | Yes | Yes |
| 3. Digital intervention element content | German | English and Swahili | Kenya & Tanzania | Single | No | Yes | Yes |
| 4. Face-to-face intervention content (incl. booklet + emergency + guide) | English | Swahili | Tanzania | Single | No | Yes | Yes |
| 5. Consent forms + information sheet | English | Swahili | Tanzania | Double + consensus | No | Yes | Yes |

**Supplementary methods 5. SPIRIT 2025 checklist of items to address in a randomized trial protocol***

| **Section / Topic** | **No** | **SPIRIT 2025 checklist item description** | **Reported on page no.** |
| --- | --- | --- | --- |
| **Administrative information** | | |  |
| Title and structured summary | 1a | Title stating the trial design, population, and interventions, with identification as a protocol | 1 |
|  | 1b | Structured summary of trial design and methods, including items from the World Health Organization Trial Registration Data Set | 2, 3 |
| Protocol version | 2 | Version date and identifier | 2 |
| Roles and responsibilities | 3a | Names, affiliations, and roles of protocol contributors | 1, 29 |
|  | 3b | Name and contact information for the trial sponsor | 28 |
|  | 3c | Role of trial sponsor and funders in design, conduct, analysis, and reporting of trial; including any authority over these activities | 28 |
|  | 3d | Composition, roles, and responsibilities of the coordinating site, steering committee, endpoint adjudication committee, data management team, and other individuals or groups overseeing the trial, if applicable | 26, 27, 28 |
| **Open science** | | |  |
| Trial registration | 4 | Name of trial registry, identifying number (with URL), and date of registration. If not yet registered, name of intended registry | 3 |
| Protocol and statistical analysis plan | 5 | Where the trial protocol and statistical analysis plan can be accessed | 21 |
| Data sharing | 6 | Where and how the individual de-identified participant data (including data dictionary), statistical code, and any other materials will be accessible | 27 |
| Funding and conflicts of interest | 7a | Sources of funding and other support (e.g., supply of drugs) | 28 |
|  | 7b | Financial and other conflicts of interest for principal investigators and steering committee members | 28 |
| Dissemination policy | 8 | Plans to communicate trial results to participants, healthcare professionals, the public, and other relevant groups (e.g., reporting in trial registry, plain language summary, publication) | 26 |
| **Introduction** | | |  |
| Background and rationale | 9a | Scientific background and rationale, including summary of relevant studies (published and unpublished) examining benefits and harms for each intervention | 3-6 |
|  | 9b | Explanation for choice of comparator | 6, 21 |
| Objectives | 10 | Specific objectives related to benefits and harms | 6 |
| **Methods: Patient and public involvement, trial design** | | |  |
| Patient and public involvement | 11 | Details of, or plans for, patient or public involvement in the design, conduct, and reporting of the trial | 7, 9, 17, Supplements |
| Trial design | 12 | Description of trial design including type of trial (e.g., parallel group, crossover), allocation ratio, and framework (e.g., superiority, equivalence, non-inferiority, exploratory) | 7, 8 |
| **Methods: Participants, interventions, and outcomes** | | |  |
| Trial setting | 13 | Settings (e.g., community, hospital) and locations (e.g., countries, sites) where the trial will be conducted | 7, 8 |
| Eligibility criteria | 14a | Eligibility criteria for participants | 8, Supplements |
|  | 14b | If applicable, eligibility criteria for sites and for individuals who will deliver the interventions (e.g., surgeons, physiotherapists) | 8, 12 |
| Intervention and comparator | 15a | Intervention and comparator with sufficient details to allow replication including how, when, and by whom they will be administered. If relevant, where additional materials describing the intervention and comparator (e.g., intervention manual) can be accessed | 7-12, Supplements |
|  | 15b | Criteria for discontinuing or modifying allocated intervention/comparator for a trial participant (e.g., drug dose change in response to harms, participant request, or improving/worsening disease) | 16, 17 |
|  | 15c | Strategies to improve adherence to intervention/comparator protocols, if applicable, and any procedures for monitoring adherence (e.g., drug tablet return, sessions attended) | 15, Supplements |
|  | 15d | Concomitant care that is permitted or prohibited during the trial | 9, 10 |
| Outcomes | 16 | Primary and secondary outcomes, including the specific measurement variable (e.g., systolic blood pressure), analysis metric (e.g., change from baseline, final value, time to event), method of aggregation (e.g., median, proportion), and time point for each outcome | 13-18 |
| Harms | 17 | How harms are defined and will be assessed (e.g., systematically, non-systematically) | 7, 17 |
| Participant timeline | 18 | Time schedule of enrollment, interventions (including any run-ins and washouts), assessments, and visits for participants. A schematic diagram is highly recommended (see Figure) | 7-10 |
| Sample size | 19 | How sample size was determined, including all assumptions supporting the sample size calculation | 21 |
| Recruitment | 20 | Strategies for achieving adequate participant enrollment to reach target sample size | 18-19 |
| **Methods: Assignment of interventions** | | |  |
| Randomization: |  |  |  |
| Sequence generation | 21a | Who will generate the random allocation sequence and the method used | 19 |
|  | 21b | Type of randomization (simple or restricted) and details of any factors for stratification. To reduce predictability of a random sequence, other details of any planned restriction (e.g., blocking) should be provided in a separate document that is unavailable to those who enroll participants or assign interventions | 19 |
| Allocation concealment  mechanism | 22 | Mechanism used to implement the random allocation sequence (e.g., central computer/telephone; sequentially numbered, opaque, sealed containers), describing any steps to conceal the sequence until interventions are assigned | 19 |
| Implementation | 23 | Whether the personnel who will enroll and those who will assign participants to the interventions will have access to the random allocation sequence | 19 |
| Blinding | 24a | Who will be blinded after assignment to interventions (e.g., participants, care providers, outcome assessors, data analysts) | 19 |
|  | 24b | If blinded, how blinding will be achieved and description of the similarity of interventions | 19 |
|  | 24c | If blinded, circumstances under which unblinding is permissible, and procedure for revealing a participant’s allocated intervention during the trial | 19 |
| **Methods: Data collection, management, and analysis** | | |  |
| Data collection methods | 25a | Plans for assessment and collection of trial data, including any related processes to promote data quality (e.g., duplicate measurements, training of assessors) and a description of trial instruments (e.g., questionnaires, laboratory tests) along with their reliability and validity, if known. Reference to where data collection forms can be accessed, if not in the protocol | 13-18 |
|  | 25b | Plans to promote participant retention and complete follow-up, including list of any outcome data to be collected for participants who discontinue or deviate from intervention protocols | 15, 18 |
| Data management | 26 | Plans for data entry, coding, security, and storage, including any related processes to promote data quality (e.g., double data entry; range checks for data values). Reference to where details of data management procedures can be accessed, if not in the protocol | 19-22 |
| Statistical methods | 27a | Statistical methods used to compare groups for primary and secondary outcomes, including harms | 21, 22 |
|  | 27b | Definition of who will be included in each analysis (e.g., all randomized participants), and in which group | 18, 21, 22 |
|  | 27c | How missing data will be handled in the analysis | 22 |
|  | 27d | Methods for any additional analyses (e.g., subgroup and sensitivity analyses) | 22 |
| **Methods: Monitoring** | | |  |
| Data monitoring committee | 28a | Composition of data monitoring committee (DMC); summary of its role and reporting structure; statement of whether it is independent from the sponsor and funder; conflicts of interest and reference to where further details about its charter can be found, if not in the protocol. Alternatively, an explanation of why a DMC is not needed | 26-27 |
|  | 28b | Explanation of any interim analyses and stopping guidelines, including who will have access to these interim results and make the final decision to terminate the trial | N/A |
| Trial monitoring | 29 | Frequency and procedures for monitoring trial conduct. If there is no monitoring, give an explanation | 26-27 |
| **Ethics** | | |  |
| Research ethics approval | 30 | Plans for seeking research ethics committee/institutional review board approval | 26 |
| Protocol amendments | 31 | Plans for communicating important protocol modifications to relevant parties | 26 |
| Consent or assent | 32a | Who will obtain informed consent or assent from potential trial participants or authorized proxies, and how | 18 |
|  | 32b | Additional consent provisions for the collection and use of participant data and biological specimens in ancillary studies, if applicable | N/A |
| Confidentiality | 33 | How personal information about potential and enrolled participants will be collected, shared, and maintained to protect confidentiality before, during, and after the trial | 19-20, 23, 27 |
| Ancillary and post-trial care | 34 | Provisions, if any, for ancillary and post-trial care, and for compensation to those who suffer harm from trial participation | 17, 27 |

*We strongly recommend reading this checklist in conjunction with the SPIRIT 2025 Explanation and Elaboration and the SPIRIT 2025 Expanded Checklist for important clarifications on all the items. We also recommend reading relevant SPIRIT extensions [22]. See [www.consort-spirit.org](http://www.consort-spirit.org)

© 2025 Chan A-W et al. This is an Open Access article distributed under the terms of the Creative Commons Attribution License (<https://creativecommons.org/licenses/by/4.0/>), which permits unrestricted use, distribution, and reproduction in any medium, provided the original work is properly cited.

**References**

1. Kaufman EA, Xia M, Fosco G, Yaptangco M, Skidmore CR, Crowell SE. The Difficulties in Emotion Regulation Scale Short Form (DERS-SF): Validation and Replication in Adolescent and Adult Samples. Journal of Psychopathology and Behavioral Assessment. 2016;38(3):443–55.

2. Burton AL, Brown R, Abbott MJ. Overcoming difficulties in measuring emotional regulation: Assessing and comparing the psychometric properties of the DERS long and short forms. Cogent Psychology. 2022;9(1):1–16.

3. Hartmann JA, Nelson B, Spooner R, Paul Amminger G, Chanen A, Davey CG, et al. Broad clinical high-risk mental state (CHARMS): Methodology of a cohort study validating criteria for pluripotent risk. Early Intervention in Psychiatry. 2019;13(3):379–86.

4. Isler J, Sawadogo NH, Harling G, Bärnighausen T, Adam M, Kagoné M, et al. Iterative adaptation of a mobile health intervention across countries using human-centered design: Qualitative study. JMIR mHealth and uHealth. 2019;7(11).

5. Adam M, McMahon SA, Prober C, Bärnighausen T. Human-centered design of video-based health education: An iterative, collaborative, community-based approach. Journal of Medical Internet Research. 2019;21(1):1–18.

6. Göttgens I, Oertelt-Prigione S. The Application of Human-Centered Design Approaches in Health Research and Innovation: A Narrative Review of Current Practices. JMIR mHealth and uHealth. 2021;9(12).

7. Bazzano AN, Martin J, Hicks E, Faughnan M, Murphy L. Human-centred design in global health_ A scoping review of applications.pdf. 2017;1–24.

8. Heim E, Kohrt BA. Cultural adaptation of scalable psychological interventions: A new conceptual framework. Clinical Psychology in Europe. 2019;1(4):0–22.

9. Myin-Germeys I, Kasanova Z, Vaessen T, Vachon H, Kirtley O, Viechtbauer W, et al. Experience sampling methodology in mental health research: new insights and technical developments. World Psychiatry. 2018;17(2):123–32.

10. Paetzold I, Schick A, Rauschenberg C, Hirjak D, Banaschewski T, Meyer-Lindenberg A, et al. A Hybrid Ecological Momentary Compassion–Focused Intervention for Enhancing Resilience in Help-Seeking Young People: Prospective Study of Baseline Characteristics in the EMIcompass Trial. JMIR Formative Research. 2022;6(11).

11. Lincoln TM, Hohenhaus F, Hartmann M. Can paranoid thoughts be reduced by targeting negative emotions and self-esteem? An experimental investigation of a brief compassion-focused intervention. Cognitive Therapy and Research. 2013;37(2):390–402.

12. Yang K, Zhang L, Li W, Jia N, Kong F. Gratitude predicts well-being via resilience and social support in emerging adults: A daily diary study. Journal of Positive Psychology. 2025;20(2):360–72.

13. Toprak B, Sarı T. The effects of a 2-week gratitude journaling intervention to reduce parental stress and enhance well-being: a pilot study among preschool parents. Discover Psychology. 2023;3(1).

14. Osborn TL, Venturo-Conerly KE, Arango G. S, Roe E, Rodriguez M, Alemu RG, et al. Effect of Shamiri Layperson-Provided Intervention vs Study Skills Control Intervention for Depression and Anxiety Symptoms in Adolescents in Kenya: A Randomized Clinical Trial. JAMA Psychiatry. 2021 Aug 1;78(8):829–37.

15. Walton GM. The New Science of Wise Psychological Interventions. Current Directions in Psychological Science. 2014;23(1):73–82.

16. Grant A, Treweek S, Dreischulte T, Foy R, Guthrie B. Process evaluations for cluster-randomised trials of complex interventions: A proposed framework for design and reporting. Trials. 2013;14(1).

17. Sicorello M, Elsaesser M, Kolar DR. Translation, Validation and Extended Factor Models of the German State Difficulties in Emotion Regulation Scale (S-DERS). PsyArXiv Preprints. 2024;

18. Medland H, De France K, Hollenstein T, Mussoff D, Koval P. Regulating Emotion Systems in Everyday Life: Reliability and Validity of the RESS-EMA Scale. European Journal of Psychological Assessment. 2020;36(3):437–46.

19. Meyer-Lindenberg A, Falkai P, Fallgatter AJ, Hannig R, Lipinski S, Schneider S, et al. The future German Center for Mental Health (Deutsches Zentrum für Psychische Gesundheit): a model for the co-creation of a national translational research structure. Nature Mental Health. 2023;1(3):153–6.

20. Guillemin F, Bombardier C, Beaton D. CROSS-CULTURAL ADAPTATION OF HEALTH-RELATED QUALITY OF LIFE MEASURES: LITERATURE REVIEW AND PROPOSED GUIDELINES. Clinical Epidemiology. 1993;46(12):1417–32.

21. WHO. Psychological Interventions Implementation Manual. 2024. 1–96 p.

22. Chan AW, Boutron I, Hopewell S, Moher D, Schulz KF, Collins GS, et al. SPIRIT 2025 statement: updated guideline for protocols of randomised trials. BMJ. 2025 Apr 28;389:e081477.
